# Effect of Adding Human Chorionic Gonadotropin to The Endometrial Preparation Protocol in Frozen Embryo Transfer Cycles

**Published:** 2012-12-17

**Authors:** Maryam Eftekhar, Elham Rahmani, Tahereh Eftekhar

**Affiliations:** 1 Department of Obstetrics and Gynecology, Yazd Research and Clinical Center for Infertility, Shahid Sadoughi University of Medical Sciences, Yazd, Iran; 2 Department of Obstetrics and Gynecology, Bushehr University of Medical Sciences, Bushehr, Iran; 3 Department of Obstetrics and Gynecology, Tehran University of Medical Sciences, Tehran, Iran

**Keywords:** ART, Implantation, Frozen Embryo, HCG

## Abstract

**Background:**

Human chorionic gonadotropin (HCG), one of the initial embryonic signals, is probably a major regulator of the embryo-endometrial relationship. This study aims to assess the advantage of HCG supplementation during the secretory phase of hormonally prepared cycles for the transfer of cryopreserved-thawed embryos.

**Materials and Methods:**

This study was a randomized clinical trial. Infertile women who were candidates for frozen-thawed embryo transfers entered the study and were divided into two groups, HCG and control. The endometrial preparation method was similar in both groups: all women received estradiol valerate (6 mg) po per day from the second day of the menstrual cycle and progesterone in oil (100 mg) intramuscular (I.M.) when the endometrial thickness reached 8 mm. Estradiol and progesterone were continued until the tenth week of gestation. In the HCG group, patients received an HCG 5000 IU injection on the first day of progesterone administration and the day of embryo transfer.

**Results:**

In this study, 130 couples participated: 65 in the HCG group and 65 in the control group. There was no statistically significant difference between groups regarding basic characteristics. Implantation rate, chemical pregnancy, clinical pregnancy, ongoing pregnancy, and abortion rates were similar in both groups.

**Conclusion:**

Although HCG has some advantages in assisted reproductive technology (ART) cycles, our study did not show any benefit of HCG supplementation during the secretory phase of frozen cycles (Registration Number: IRCT201107266420N4).

## Introduction

Implantation is a complex, important process (1 ). Embryo quality and uterine receptivity are important factors that influence the outcome of assisted reproductive technology (ART). Various substances such as cyclic adenosine monophosphate (cAMP), relaxin, gonadotropin, prostaglandin E2 (PGE2), and glycoprotein hormones that are secreted from the embryo or endometrium affect implantation (2 -7 ). Secretion of human chorionic gonadotropin (HCG) is one of the initial embryonic signals. It is probably a major regulator of the embryo-endometrial relationship. Expression of the HCG/luteinizing hormone (HCG/ LH) receptor is observed in the endometrium during the secretory phase of menstrual cycles (7 ). HCG is usually defined as a key factor that can induce and develop *in vitro* decasualization of the endometrium (6 , 8 ). HCG may have systemic and local effects on the embryo-endometrial microenvironment (9 ).

Pregnancy rates following cryopreserved embryo transfer cycles are usually lower than fresh embryo transfer cycles. However, transfer of excess embryos in frozen cycles increases the cumulative pregnancy rates and decreases the costs. So an attempt to evaluate the factors that influence the success rate of cryopreserved cycles is important (9 , 10 ). The aim of this study is to assess the advantage of HCG supplementation during the secretory phase of hormonally-prepared cycles for the transfer of cryopreserved-thawed embryos.

## Materials and Methods

This randomized clinical trial was conducted at the Yazd Research and Clinical Center for Infertility, Shahid Sadoughi University of Medical Sciences between January 2009 and June 2011. The study was approved by the Ethics Committee of the university. Written informed consent was obtained from all couples. Women who had undergone *in vitro* fertilization (IVF) or intracytoplasmic sperm injection (ICSI) with cryopreservation of excess embryos and fresh cycles with implantation failure entered the study. Women older than 38 years, those with a body mass index (BMI) >30 kg/m^2^, history of endocrine disorders, and severe endometriosis were excluded from the study. The patients were allocated into two groups: group 1 (HCG) and group 2 (control) as performed by computerized randomization.

Morphological assessment of all embryos was performed on the second day after oocyte retrieval. We transferred two or three embryos. Any excess embryos that had less than 30% fragmentation were frozen by the vitrification method. After a two-step loading with equilibrant and vitrification solutions, embryos were loaded by a thin glass capillary on to the cryoton. After loading, nearly the entire solution was eliminated and only a fine layer covering the embryos remained. The embryos were flooded in to liquid nitrogen, a film part of cryoton was covered by a plastic cap, and we stored the sample in liquid nitrogen. Thawing was performed at least two months after cryopreservation, when straws were exposed to air and then submerged in water. The cryoprotectant was eliminated using embryo-thawing media (Vitrolife). After thawing, the embryos were transferred to a culture media and evaluated 24 hours later. We selected those embryos with less than 50% fragmentation for transfer.

The endometrial preparation process was similar in both groups. All women received oral estradiol valerate (2 mg, Aburaihan Co., Tehran, Iran) 6 mg per day from the second day of the menstrual cycle. Endometrial thickness was measured by vaginal ultrasonography. From the 13^th^ day of the cycle when endometrial thickness reached 8 mm, intramuscular (IM) injections of progesterone in oil (100 mg; Aburaihan Co., Tehran, Iran) were administered to all subjects. Embryo transfer was performed three days after the beginning of progesterone administration. Estradiol and progesterone were continued until the tenth week of gestational age.

In the HCG group, 5000 IU HCG (Pregnyl®, Organon, Oss, Netherlands) was injected on the first day of progesterone administration and on the day of embryo transfer. Embryo thawing was performed two days after the first progesterone injection. Embryos were transferred one day after thawing by a Labotect catheter (Labotect, Gotting, Germany).

Chemical pregnancy was defined by serum β hCG >50 IU/L 12 days after embryo transfer and clinical pregnancy was defined by observation of fetal heart activity two weeks after positive β hCG. We defined miscarriage as the loss of pregnancy before the 20^th^ week of gestation and ongoing pregnancy as continuation of pregnancy after the 12^th^ week of gestation. Implantation rate was defined as the numbers of gestational sacs per 100 embryos transferred.

### Statistical analysis

Statistical analysis was carried out using the statistical package for the social sciences (SPSS version 15.0 for Windows, Chicago, IL). Both t test and chi-square test were used to detect significant differences between two groups. The level of significance was set at p value<0.05.

### Results

In this study, a total of 130 couples participated: 65 in group 1 (HCG group) and 65 in group 2 (control group). The demographic and basic characteristics of patients are shown in table 1.

There were no statistically significant differences between groups regarding age (p=0.549), duration of infertility (p=0.368), basal follicle stimulating hormone (p=0.135), BMI (p=0.661), and etiology of infertility (p=0.201). The cycle characteristics and outcome of vitrification are shown in table 2.

There were no statistically significant differences between groups regarding the numbers of thawed embryos, numbers of transferred embryos, survival rates of thawing embryos and duration of freezing. Table 3 shows the outcome of ART cycles. Implantation, chemical pregnancy, clinical pregnancy, ongoing pregnancy, and abortion rates were similar in both groups.

**Table 1:** Basic patient characteristics in the two groups


Variables	HCG group Mean (SD)	Control group Mean (SD)	P value

**Age (Years)**	28.47 ± 4.14	28.84 ± 3.71	0.549
**Duration of infertility (Years)**	6.58 ± 2.9	6.09 ± 2.74	0.368
**Basal FSH (IU/L)**	5.15 ± 1.66	5.60 ± 1.72	0.135
**BMI (kg/m^2^)**	23.67 ± 2.43	23.86 ± 2.3	0.661
**Etiology of infertility**			
**Ovulatory , n (%)**	13 (20)	13 (20)	0.201
**Tubal, n (%)**	9 (13.8)	10 (15.4)	
**Unexplained, n (%)**	0 (0.0)	4 (3.1)	
**Mixed, n (%)**	42 (64.6)	35 (53.8)	
**Male, n (%)**	1 (1.5)	4 (3.1)	
**Total, n (%)**	63 (100)	65 (100)	


**Table 2:** Cycle characteristics and outcome of vitrification


Variables	HCG group Mean (SD)	Control group Mean (SD)	P value

**Duration of freezing (Months)**	6.58 ± 3.1	6.09 ± 2.74	0.326
**Number of thawed embryos**	2.96 ± 0.17	2.89 ± 0.31	0.086
**Survival rate after thawing (%)**	91.79 ± 0.20	94.87 ± 0.12	0.299
**Numbers of transferred embryos**	2.73 ± 0.44	2.60 ± 0.49	0.095


**Table 3:** ART outcome in both groups


Variables	HCG group	Control group	P Value

**Implantation rate (%)**	21.02	17.44	0.488
**Chemical pregnancy rate, n (%)**	27 (41.5)	25 (38.5)	0.429
**Clinical pregnancy rate, n (%)**	22 (30.8)	20 (30.8.7)	0.426
**Ongoing pregnancy rate, n (%)**	20 (30.8)	18 (27.7)	0.424
**Miscarriage rate, n (%)**	7 (25.9)	7 (28.0.4)	0.556
**Endometrial thickness (mm)**	8.83±1.6	9.08±1.2	0.528


### Discussion

While treatment protocols, early embryo development, and laboratory techniques have considerably improved over the last decades in ART cycles, little has been identified about the events that occur after the transfer of embryos in to the uterine cavity. About 75% of embryos do not implant after transfer. Thus, to improve implantation rates we need additional understanding of the molecular mechanisms governing the endometrial preparation for embryo implantation (11 -13 ).

According to our data, for better embryo implantation it is necessary to have a good embryo with appropriate morphology and developmental potential as well as a high concentration of HCG. In this study we have hypothesized that HCG supplementation in the secretory phase of the endometrium during the frozen embryo cycle may increase the implantation and eventually the pregnancy rate. However our findings have contradicted with this hypothesis.

Our study showed that HCG supplementation for endometrial preparation in cryopreserved cycles had no more benefit than estradiol and progesterone. Our finding was consistent with the Ben-Meir et al. study where the researchers observed that recombinant HCG supplementation during the secretory phase of the frozen-thawed embryo transfer cycles showed no advantage in terms of pregnancy and implantation rates (9 ). Similar to our study, they did not use a GnRH agonist for down-regulation, and thus endogenous basal LH secretion was not completely suppressed. Tesarik et al. (14 ) demonstrated that HCG supplementation during the luteal phase of the oocyte donation cycle improved pregnancy rates only in cycles with low endogenous LH.

Mansour et al. (15 ) have shown that intrauterine injection of 500 IU HCG prior to embryo transfer significantly increased pregnancy rate after ART. They concluded that the HCG level positively correlated with the level of trophoblastic tolerance.

HCG plays a central role in controlling implantation and early embryonic development (16 ). This hormone is produced very early by the developing embryo and is secreted in relatively high concentrations. HCG may effectively regulate endometrial preparation via the following processes (17 ): i. local down-regulation of insulin growth factor binding protein 1 (IGF BP-1) by HCG leads to the prolongation of the window of endometrium; ii. HCG augments the endometrial receptivity by increasing angiogenesis through increased local vasoendothelial growth factor (VEGF) (18 , 19 ); iii.HCG interacts with the production of galactosemic fibroblast (M-GSF) and leukemia inhibiting factor (LIF) that are important for implantation (20 ); iv. HCG significantly inhibits the production of metalloproteinase (21 ); and v. HCG has been demonstrated to have an impact on the trophoblasts, resulting in improved differentiation and invasion potential (7 , 22 ).

Fatemi et al. have concluded that the spontaneous natural cycle is superior to the HCG induced natural cycle. They showed a negative effect of HCG administration on pregnancy. These researchers hypothesized that the significantly lower pregnancy rate in the HCG group was related to desynchronization between a receptive endometrium and embryo, which resulted from the endometrial effects of HCG. Administration of HCG has been shown to induce a series of events in the endometrium which begins several days later. These events may have a negative impact on implantation (23 ). Additional studies with larger sample sizes are required for better evaluation.

## Conclusion

While HCG has some advantages in the ART cycle, our study did not show any advantage of HCG supplementation in the secretory phase of frozen cycles.
