# Effect of Adding Human Chorionic Gonadotropin to The Endometrial Preparation Protocol in Frozen Embryo Transfer Cycles

**DOI:** 10.22074/IJFS.2021.244054

**Published:** 2021-10-16

**Authors:** Maryam Eftekhar, Elham Rahmani, Tahereh Eftekhar

**Affiliations:** 1 Department of Obstetrics and Gynecology, Yazd Research and Clinical Center for Infertility, Shahid Sadoughi University of Medical Sciences, Yazd, Iran; 2 Department of Obstetrics and Gynecology, Bushehr University of Medical Sciences, Bushehr, Iran; 3 Department of Obstetrics and Gynecology, Tehran University of Medical Sciences, Tehran, Iran

## Abstract

In this article which was published in Int J Fertil Steril , Vol 6, No 3, Oct-Dec 2012, on Pages: 175-178, the authors found that Four samples from the control group were incorrectly included in the study. 4 cases were removed from the data and the data were re-analyzed. The results in Tables 1-3 are corrected. The authors would like to apologies for any inconvenience caused.

## Results

A total of 126 couples participated: 65 in group 1 (HCG group) and 61 in group 2 (control group). The demographic and basic characteristics of patients are shown in Table 1.

There were no statistically significant differences between groups regarding age (P=0.696), duration of infertility (P=0.222), basal follicle stimulating hormone (P=0.061), BMI (P=0.526), and etiology of infertility (P=0.294). The cycle characteristics and outcome of vitrification are shown in Table 2. 

There were no statistically significant differences between groups regarding the numbers of thawed embryos, numbers of transferred embryos, survival rates of thawing embryos and duration of freezing. Table 3 shows the outcome of ART cycles. Implantation, chemical pregnancy, clinical pregnancy, ongoing pregnancy, and abortion rates were similar in both groups.

**Table 1:** Basic patient characteristics in the two groups


Variables	HCG group	Control group	P value

Age (Y)	28.47 ± 4.14	28.74 ± 3.55	0.696
Duration of infertility (Y)	6.58 ± 2.9	5.96 ± 2.68	0.222
Basal FSH (IU/L)	5.15 ± 1.66	5.72 ± 1.70	0.061
BMI (kg/m^2^)	23.67 ± 2.43	23.95 ± 2.39	0.526
Etiology of infertility			
Ovulatory	13 (20)	11 (18)	0.294
Tubal	9 (13.8)	10 (16.4)	
Unexplained	0 (0.0)	3 (4.9)	
Mixed	42 (64.6)	34 (55.7)	
Male	1 (1.5)	3 (4.9)	
Total	65 (100)	61 (100)	


Data are presented as mean ± SD or n (%). HCG; Human chorionic gonadotropin, FSH; Follicle-stimulating hormone, and BMI; Body mass index.

**Table 2:** Cycle characteristics and outcome of vitrification


Variables	HCG group	Control group	P value

Duration of freezing (months)	6.58 ± 3.1	6.18 ± 2.74	0.445
Number of thawed embryos	2.96 ± 0.17	2.88 ± 0.32	0.068
Survival rate after thawing (%)	91.79 ± 0.20	94.54 ± 0.12	0.369
Numbers of transferred embryos	2.73 ± 0.44	2.57 ± 0.49	0.052


Data are presented as mean ± SD. HCG; Human chorionic gonadotropin.

**Table 3:** ART outcome in both groups


Variables	HCG group	Control group	P value

Implantation rate (%)	21.02	17.44	0.488
Chemical pregnancy rate, n (%)	27 (41.5)	25 (41.0)	0.950
Clinical pregnancy rate, n (%)	22 (33.8)	20 (32.8)	0.900
Ongoing pregnancy rate, n (%)	20 (30.8)	18 (27.7)	0.878
Miscarriage rate, n (%)	7 (25.9)	7 (28.0)	0.866
Endometrial thickness (mm)	8.83 ± 1.6	9.08 ± 1.10	0.427


Data are presented as mean ± SD or n (%) or %. HCG; Human chorionic gonadotropin, ART; Assisted reproductive technology.

